# Health-related quality of life after cardiac surgery – the effects of age, preoperative conditions and postoperative complications

**DOI:** 10.1186/1749-8090-9-46

**Published:** 2014-03-11

**Authors:** Vojtěch Kurfirst, Aleš Mokráček, Martina Krupauerová, Júlia Čanádyová, Alan Bulava, Ladislav Pešl, Věra Adámková

**Affiliations:** 1 Cardiac Surgery Department, Boženy Němcové str. 54, Hospital České Budějovice, České Budějovice, Czech Republic; 2 KardioECHO Cardiology Outpatients Department, České Budějovice, Czech Republic; 3 Department of Cardiology, Hospital České Budějovice, České Budějovice, Czech Republic; 4 Faculty of Health and Social Studies, University of South Bohemia, České Budějovice, Czech Republic; 5 Preventive Cardiology Department, Institute for Clinical and Experimental Medicine, Prague, Czech Republic

**Keywords:** Health-related quality of life, Postoperative complications, Older patients, Cardiac surgery

## Abstract

**Background:**

Factors influencing the postoperative health-related quality of life (HRQOL) after cardiac surgery have not been well described yet, mainly in the older people. The study’s aim was to explore differences in clinical conditions and HRQOL of patients before and after cardiac surgery taking into account the influence of age and to describe factors influencing changes of HRQOL in the postoperative period.

**Methods:**

This was a prospective consecutive observational study with two measurements using the SF-36 questionnaire before surgery and 1 year after surgery. It considered main clinical characteristics of participants prior to surgery as well as postoperative complications.

**Results:**

At baseline assessment the study considered 310 patients, predominantly male (69%). Mean age was 65 (SD 10.4) years and 101 patients (33%), who were older than 70, constituted the older group. This older group showed greater comorbidity, higher cardiac operative risk and lower HRQOL in the preoperative period as well as a higher prevalence of postoperative complications than the younger group. Thirty-day mortality was 1.4% in the younger group and 6.9% in the older group (p < 0.001). One year mortality was 3.3% in the younger group and 10.9% in the older group (p < 0.001). There was a significant improvement in all 8 health domains of the SF-36 questionnaire (p < 0.001) in the overall sample. There was no significant difference in change in a majority of HRQOL domains between the younger and the older group (p > 0.05). Logistic multivariate analysis identified a higher values of preoperative PCS (Physical component summary) scores (OR 1.03, CI 1.00 – 1.05, p = 0.0187) and MCS (Mental component summary) scores (OR 1.02, CI 0.997 – 1.00, p = 0.0846) as the only risk factors for potential non-improvement of HRQOL after cardiac surgery after correction for age, gender and type of surgery.

**Conclusions:**

Older patients with higher operative risk have lower preoperative HRQOL but show a similar improvement in a majority of HRQOL domains after cardiac surgery as compared with younger patients. The multivariate analysis has shown the higher preoperative HRQOL status as a only significant factor of potential non-improvement of postoperative HRQOL.

## Background

Health-related quality of life (HRQOL) is an increasingly important aspect in assessing the outcome of any surgical intervention [1]. Simultaneously life expectancy has increased, particularly in recent decades, thus leading to an increase in the numbers of an aging population undergoing cardiac surgery [2]. Age is an independent factor in calculating the risk of death during cardiac surgery [3,4]. Because older patients usually suffer from comorbidities which can make the course of an operation significantly longer [5], and also because they have lower functional reserves [6], there is a higher prevalence of postoperative complications or death. Studies concerning the influence of age on postoperative HRQOL have produced differing conclusions. While some have presented better postoperative HRQOL outcomes for younger patients [7], others have found the opposite [8]. The influence of age on HRQOL has not yet been definitively demonstrated.

In the postoperative period, the most frequent complications has been described as supraventricular arrhythmia, infection, cognitive impairment, as well as respiratory and renal insufficiency [9]. Older patients are also given more units of red blood cell transfusion, which corresponds to a higher incidence of preoperative anemia, smaller capacity of the hematopoietic system, and a lower tolerance of postoperative hypohemoglobinaemia [10]. Providing a greater number of red cell units has been identified as an independent factor in postoperative mortality [11]. Although these complications have been well described, there is still not enough information about the impact of postoperative complications on HRQOL.

The SF-36 questionnaire is often used as a tool for assessing the quality of life in various medical fields, where it is valued especially for its ability to capture the social dimensions of life [12]. In cardiology, for example, it has been used in patients with ischaemic heart disease [13], in the course of heart failure [14], or in the presence of atrial fibrillation [15]. In cardiac surgery, an assessment of HRQOL by means of the SF-36 questionnaire has been used in patients undergoing myocardial revascularization [16], after surgery on heart valves [17,18] or the thoracic aorta [19], and after interventions for cardiac arrhythmias [20]. It also has proven effective in patients after valve replacement with a mechanical heart valve prosthesis which when closing produces a clicking sound [21].

The aim of this study was to assess the HRQOL of two groups of patients (age ≤70 and >70 years) by using the SF-36 questionnaire and to compare clinical data from the preoperative, perioperative, and postoperative periods experienced by these groups. A second aim was to identify a group of patients who do not benefit from the surgery (non-improvers) and to find potential risk factors for unimproved quality of life after cardiac surgery.

## Methods

The study was carried out with the approval of the Ethics Committee of the Hospital of České Budějovice. In the period from January 2008 to June 2009, 873 patients were operated on in the Cardiac Surgery Department of Hospital České Budějovice. All patients included in this study were operated on by a single surgeon, and the total number of patients was 310. All procedures were performed in a standard manner using extracorporeal circulation and during heart arrest. No off-pump procedures were used in the study population. The grafts used during revascularization procedures were LIMA (left internal mammary artery) and vein grafts. The inclusion criteria were: elective cardiac surgery, agreement with participation in the study, and written informed consent. Patients undergoing urgent cardiac surgery and patients who refused to participate in the study were excluded. SF-36 questionnaires were completed before surgery and 1 year after discharge from the hospital. All patients participating in the study had agreed to do so, and written informed consent was obtained.

The SF-36 questionnaire was used to evaluate HRQOL of patients included in this study. Evaluation of HRQOL using the SF-36 is based on 8 domains covering the physical, mental, and social life of the individual. These domains are as follow: Physical functioning, Role physical, Role emotional, Social functioning, Bodily pain, Mental health, Vitality and General health. Questions are related to the period 4 weeks before completion of the questionnaire, and therefore the evaluation is not influenced by transitory changes in status. Answers are then converted to 0-100 scale for each health domain where higher values indicate better health status. A standardized procedure is used for interpreting responses given to the SF-36 questionnaire [22,23].

Clinical data from the preoperative, perioperative and postoperative period were collected from medical records. All clinical variables and events were defined according to standard European Society of Cardiology definitions.

The differences between the preoperative and postoperative HRQOL were assessed using the non-parametric Wilcoxon matched-pairs signed-rank test. The total number of patients was divided into two groups (age ≤70 and >70 years) and an HRQOL assessment was performed 1 year after surgery, after which we analyzed and compared the two groups of patients. We also compared HRQOL between survivors and non-survivors. The differences between subgroups were compared by unpaired t-test and χ^2^ test.

For all subgroups, p-values ≤ 0.05 were considered statistically significant. Multivariate analysis was performed using a logistic regression model to examine the association between preoperative variables (preoperative PCS and MCS, age, gender, type of surgery) and postoperative HRQOL improvement. Improvement was defined as a positive increase in postoperative SF-36 subscales.

Physical component summary (PCS) and mental component summary (MCS) scores were used for the multivariate analysis. The PCS and MCS scores are two meta-scores of the SF-36 calculated from the SF-36 questionnaire and reflect a patient’s overall physical and mental health status. PCS is consisted of these domains of SF-36 questionnaire: Physical functioning, Role physical, Bodily pain, Vitality and General health. MCS is consisted of these domains: Role emotional, Social functioning and Mental health. These summary scales were used in this study as the primary health-related HRQOL variable for the multivariate analysis. Non-improvers were defined as patients with difference ≤ 0 between preoperative and postoperative quality of life in both PCS and MCS scores. Improvers were defined as patients with difference > 0 between preoperative and postoperative quality of life in PCS or MCS or both scores.

Patients’ risk scores were obtained via the EuroSCORE calculator, which is free accessible at the web site http://www.euroscore.org. The mortality data were obtained via the registry of the Czech Society of Cardiovascular Surgery. Statistical analyses were performed using STATISTICA 10 [24] and XLSTAT version 2011 [25].

## Results

Preoperative characteristics of the 310 patients are summarized in Table 1. Men accounted for 69% of the patients, and the mean age was 65 ± 10.4 years. In the preoperative period, the older group showed a higher prevalence of hypertension (p = 0.001), diabetes (p = 0.026), atrial arrhythmia (p < 0.001), renal dysfunction (p = 0.008), cerebrovascular disease (p = 0.045), and anemia (p = 0.027). Their collective EuroSCORE was also higher (p < 0.001). There were no statistical differences in the durations of cardiopulmonary bypass, aortic cross-clamp, or anesthesia times between the two groups of patients (Table 2).

**Table 1:** Preoperative clinical characteristics of all patients

	**Age ≤70 years**			**Age >70 years**			**p**
**Variables**	**No.**	**Mean**	**%**	**No.**	**Mean**	**%**	
Total	209		67.4	101		32.6	
Male	156		74.6	56		55.4	0.001
Age (years)		59.9			75.4		<0.001
Reoperation	11		5.3	4		4.0	0.617
Prior PCI	35		16.7	11		10.9	0.174
History of MI	31		14.8	12		12.9	0.771
Hypertension	126		60.3	80		79.2	0.001
Diabetes mellitus	53		25.4	38		37.6	0.026
Supraventricular arrhythmia	26		12.4	33		32.7	<0.001
COPD	27		12.9	8		7.9	0.192
Renal dysfunction	25		12.0	24		23.8	0.008
Cerebral vascular disease	15		7.2	14		13.9	0.058
History of CVA	10		4.8	11		10.9	0.045
PAD	8		3.8	4		4.0	0.100
Anemia	9		4.3	11		10.9	0.027
EuroSCORE		4.3			10.5		<0.001
LVEF (%)		59.6			60.9		0.372
Age of patients not included in the QoL assessment		61.1			76.3		<0.001
EuroSCORE of patients not included in the QoL assessment		3.8			11.2		<0.001

No. – number, Reoperation – previous cardiac operation, PCI – percutaneous coronary intervention, MI – myocardial infarction, Diabetes mellitus – treated by subcutaneous insulin or by oral antidiabetics, Supraventricular arrhythmia – atrial fibrillation or atrial flutter, COPD – chronic obstructive pulmonary disease, Renal dysfunction – serum creatinine ≥ 120 umol/l, Cerebral vascular disease – stenosis of carotid artery >70%, CVA – cerebrovascular accident, PAD – peripheral artery disease, Anemia – serum hemoglobin < 12 g/dL (women) or < 13 g/dL (men), LVEF – left ventricular ejection fraction.

**Table 2:** Perioperative clinical characteristics of the studied patients

**Variables**	**Age ≤70 years**				**Age >70 years**				**p**
	**No.**	**Mean**	**%**	**SD**	**No.**	**Mean**	**%**	**SD**	
Coronary artery bypass grafting	73		34.9		39		38.6		<0.001
Valve procedures	75		35.9		25		24.8		<0.001
Combined procedures	61		29.2		37		36.6		0.038
Cardiopulmonary bypass time (min)		87.2		41.5		88.4		30.1	0.795
Aortic cross-clamp time (min)		62.0		35.9		59.3		22.4	0.495
Anesthesia time (min)		255.2		55.4		259.7		51.9	0.505

In the postoperative period, the prevalences of cerebrovascular events (p = 0.001), renal failure (p = 0.018), infection (p = 0.005), and cognitive impairment (p = 0.006) were also higher in the older group (Table 3). The number of packed red blood cell units given in the postoperative period was higher in the older group (p = 0.012). Thirty-day mortality was 1.4% (3 patients) in the younger group and 6.9% (7 patients) in the older group (p < 0.001). One year mortality was 3.3% (7 patients) in the younger group and 10.9% (11 patients) in the older group (p < 0.001). The median length of ICU/hospital stay was 2/7 days in the younger group and 2/8 days in the older group.

**Table 3:** Postoperative complications and additionally assessed variables

	**Age ≤70 years**			**Age >70 years**			**p**
**Variables**	**No.**	**Mean**	**%**	**No.**	**Mean**	**%**	
Inotropic drug support required	81		38.9	44		43.4	0.446
MI	4		1.9	1		1.0	0.543
CVA	10		0.5	7		6.9	0.001
Supraventricular arrhythmia	88		42.1	51		50.5	0.164
Ventricular arrhythmia	7		3.3	2		2.0	0.502
Renal failure	7		3.3	10		9.9	0.018
Infection	21		10.0	22		21.8	0.005
Reexploration for bleeding	7		3.3	3		3.0	0.862
Cognitive impairment	17		8.1	19		18.8	0.006
Ventilation problems	10		4.8	8		7.9	0.269
Sternal wound infection	8		3.8	4		4.0	1.000
Packed red blood cells (units)		2.1			3.1		0.012
Platelets (units)		0.3			0.2		0.731
Fresh frozen plasma (units)		1.6			2.4		0.090
Median ICU stay time (days)	2 (1 – 27)			2 (1 – 32)			0.095
Median hospital stay time (days)	7 (4 – 41)			8 (4 – 44)			0.661

MI – myocardial infarction, CVA – cerebrovascular accident, Supraventricular arrhythmia – atrial fibrillation or atrial flutter, Ventricular arrhythmia – ventricular tachycardia or fibrillation, Renal failure – increase of serum creatinine > 80 mmol/l with or without need for temporary dialysis, Infection – infection of respiratory or urinary system, Cognitive impairment – psychomotor agitation with the necessity of therapeutic intervention, Ventilation problems – symptoms of partial and/or global respiratory failure with or without need for reintubation, ICU – intensive care unit.

Completed SF-36 questionnaires 1 year after surgery were obtained from 260 patients (84%) and used for the statistical assessment. Incomplete SF-36 questionnaires from 19 patients as well as unreturned questionnaires from another 31 patients (18 patients died in the study period, 13 patients did not return the questionnaire) were not considered in the statistical assessment. The characteristics of the patients returning incomplete SF-36 questionnaires or not returning the questionnaire are given in Table 1. The results of changes in preoperative and postoperative SF-36 scores are presented in Table 4. Postoperative SF-36 scores of the study group significantly improved in all 8 health domains: Physical functioning, Role physical, Bodily pain, General health, Vitality, Social functioning, Role emotional, and Mental health. Next, patients were divided into two groups according to their age (age ≤70 years and >70 years). The preoperative SF-36 scores for HRQOL were relatively higher for the younger group (Table 5), and we found relatively greater differences between preoperative and postoperative HRQOL domains in the group age >70 years. The only statistically significant difference between the younger and the older groups, however, was in the perception of *bodily pain* domain(p = 0.03), which improved more in the older group of patients. Comparison of postoperative SF-36 results between these two groups is presented in Table 6.

**Table 4:** SF-36 scores of the study population before and one year after cardiac surgery

	**Before surgery**		**One year after surgery**		***p***
	**n = 260**		**n = 260**		
**Sub-score**	**Mean**	**SD**	**Mean**	**SD**	
PF	48.2	28.1	65.5	24.1	<0.001
RP	28.8	37.3	51.1	38.8	<0.001
BP	59.1	25.9	73.9	23.4	<0.001
GH	46.9	17.2	51.8	17.9	<0.001
VT	48.3	19.9	57.4	18.9	<0.001
SF	66.1	22.1	73.6	22.7	<0.001
RE	42.6	42.3	67.9	35.3	<0.001
MH	59.7	20.3	70.6	16.5	<0.001

PF – physical functioning, RP – role physical, BP – bodily pain, GH – general health, VT – vitality, SF – social functioning, RE – role emotional, MH – mental health.

**Table 5:** SF-36 scores of patient groups age ≤70 and >70 years before cardiac surgery

	**Age ≤70 years**		**Age >70 years**		***p***
	**n = 179**		**n = 81**		
**Sub-score**	**Mean**	**SD**	**Mean**	**SD**	
PF	54.2	28.1	35.1	25.4	<0.001
RP	34.2	39.3	16.7	29.6	<0.001
BP	62.7	26.6	51.3	22.8	<0.001
GH	48.6	17.8	43.1	15.4	<0.001
VT	50.6	21.0	43.1	16.5	<0.001
SF	68.3	22.4	61.3	21.0	<0.001
RE	48.2	43.3	30.0	37.1	<0.001
MH	62.3	20.4	54.9	19.4	<0.001

PF – physical functioning, RP – role physical, BP – bodily pain, GH – general health, VT – vitality, SF – social functioning, RE – role emotional, MH – mental health.

**Table 6:** Differences in SF-36 scores before and 1 year after cardiac surgery between patient groups age ≤70 years and >70 years

	**Age ≤70 years**		**Age >70 years**		***p***
	**n = 179**		**n = 81**		
**Sub-score**	**Difference**	**SD**	**Difference**	**SD**	
PF	15.4	29.2	21.2	27.4	0.13
RP	20.7	47.6	25.9	48.8	0.41
BP	11.9	31.4	21.2	32.7	0.03
GH	4.2	22.1	6.4	17.6	0.43
VT	8.4	23.5	10.8	22.1	0.43
SF	6.2	27.3	10.2	26.3	0.27
RE	22.9	50.3	30.9	47.7	0.23
MH	9.7	21.7	13.7	21.7	0.17

PF – physical functioning, RP – role physical, BP – bodily pain, GH – general health, VT – vitality, SF – social functioning, RE – role emotional, MH – mental health.

**Figure 1:**
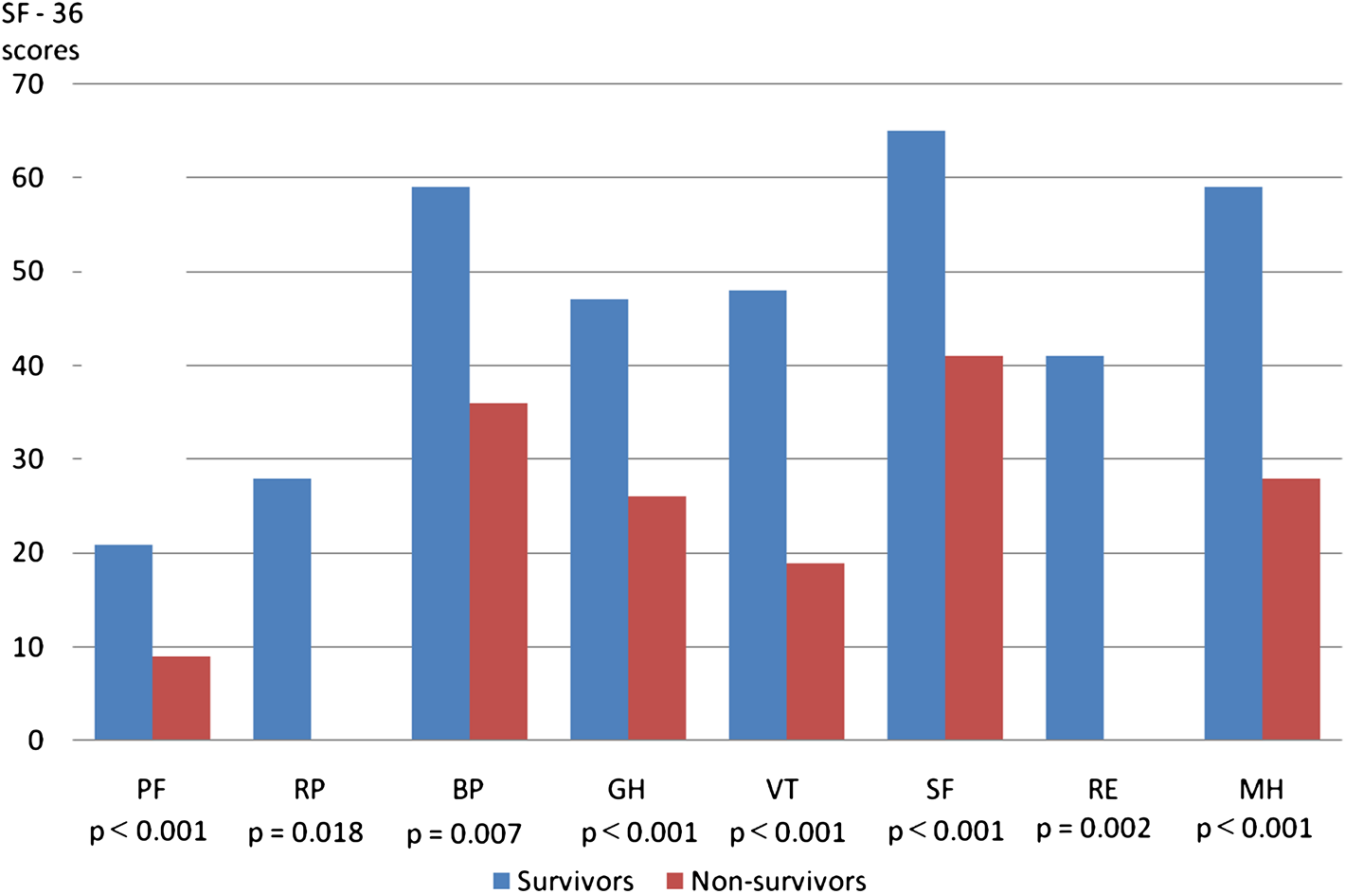
SF-36 differences in preoperative HRQOL between survivors and non-survivors.

When comparing the subgroups of survivors and non-survivors, we found significant differences in all 8 HRQOL domains of the preoperative SF-36 questionnaire (Figure 1). There were 16% of non-improvers, 10% of patients improved only in PCS scores, 9.5% of patients improved only in MCS scores and 64% of patients in our group improved in both PCS and MCS scores of HRQOL. The characteristics of improvers and non-improvers are listed in Table 7. The results from multivariate analysis demonstrate that among the preoperative variables only preoperative HRQOL status was a significant factor associated with potential non-improvement in postoperative quality of life (Table 8). The highest risk of non-improvement was found in those patients having higher preoperative PCS and MCS scores.

**Table 7:** Characteristics of improvers and non-improvers group

	**Improvers**		**Non-improvers**		
	**n = 218**		**n = 42**		
**Variables**	**Mean**	**SD**	**Mean**	**SD**	**p-value**
Ejection fraction (%)	61.0	11.4	61.2	11.5	0.93
Age	65.6	9.5	63.3	10.0	0.17
BMI	29.3	4.6	30.0	5.2	0.4
ICU stay time (days)	3.2	2.8	3.3	1.9	0.75
Hospital stay time (days)	9.1	5.4	11.5	8.8	0.09
Preoperative PCS	40.9	23.6	66.5	23.7	<0.001
Postoperative PCS	66.5	23.3	46.2	20.2	<0.001
Preoperative MCS	52.1	23.2	74.9	20.0	<0.001
Postoperative MCS	73.9	18.8	54.1	22.4	<0.001

BMI – Body mass index, ICU – Intensive care unit, PCS – Physical component summary, MCS – Mental component summary.

**Table 8:** Multivariate analysis of influence of preoperative PCS and MCS scores on postoperative quality of life improvement

**Variable**	**Regression coefficient**	**Odds ratio**	**Confidence interval (95%)**	**P value**
Gender				0.3155
Age >70 years				0.1103
Type of surgery				0.1931
Preoperative PCS	0.026	1.03	1.00 – 1.05	0.0187
Preoperative MCS	0.022	1.02	0.997 – 1.00	0.0846
Constant	- 4.415			

PCS – Preoperative physical component summary, MCS – Preoperative physical component summary; in brackets are P values for preoperative MCS status.

## Discussion

The main finding of our study is coming up from the multivariate analysis, where the given variables (age, gender, type of surgery, preoperative PCS and MCS) were tested on their influence on potential non-improvement of HRQOL after cardiac surgery. The multivariate analysis has demonstrated, that only the preoperative PCS and MCS status has influence on postoperative change in HRQOL after cardiac surgery. The highest risk of non-improvement in postoperative quality of life was found in patients with higher preoperative PCS and MCS scores. We have also found that non-survivors showed a significantly lower preoperative HRQOL than survivors. Rumsfeld et al. analyzed a set of patients undergoing myocardial revascularization and compared two components of the SF-36 questionnaire (mental and physical) in patients who survived and did not survive the first 6 months after surgery [26]. The results indicate that the preoperative health status was significantly different in the HRQOL of survivors and was the major determinant of change in quality of life following surgery. Factors presented as being associated with failure to achieve a better HRQOL outcome in the postoperative period include the following: one or more preoperative comorbidities and postoperative low cardiac output [27], low preoperative ejection fraction [28], preoperative ICU stay or perioperative complications [29], a higher dyspnea classification, experiencing a new cardiac arrhythmia during or after the surgery, higher pulmonary pressure, previous cardiac surgery, previous myocardial infarction, and manual occupation [14,30]. Based on these results we can conclude, that patients with distinctly low preoperative HRQOL status are also in very poor clinical conditions and the risk of death is increased. On the other hand, patients with high pre-surgical HRQOL don´t have much room for improvement and their surgery is more preventive in nature.

The most frequent postoperative complications in the group of older patients were previously described as heart failure, dysrhythmia, postoperative bleeding, ventilation problems, neurophysical disorders, myocardial dysfunction, and renal failure [6]. In our study, there were also higher incidences of respiratory and urinary tract infections and higher numbers of red cell units given in the group of patients age >70 years. Engoren et al. describe the same situation between groups of septuagenarians and octogenarians, and they also report hospital costs 35% higher for the octogenarians because of postoperative complications [11]. In another study, Dumbor et al. take into account the economic dimension and refer to several factors which increase hospital costs: high preoperative risk as determined by scoring systems, unintended procedures, the total red cell units used, invasive monitoring, prolonged postoperative ventilation, length of stay in ICU, incidence of postoperative atrial fibrillation and infection, and overall length of hospital stay. According to their calculations, the costs of treatment for patients age >70 years are 91% higher than are those for patients age ≤70 years [31]. Frelich et al. mention 15% higher hospital costs in patients age >70 years [6]. In the present situation, when the economic view on medical care is projecting more and more into everyday practice, our efforts should be oriented toward these high-risk patients because most of the complications are related to preoperative status and can be reduced through careful preoperative conditioning, gentle operating techniques, and appropriate postoperative care. In cardiac surgery, as in other fields, there continue to be developed new operating methods (e.g., minimally invasive) that are directed to older patients and which decrease the risk of postoperative complications and mortality while also reducing economic costs [32].

The HRQOL improves early after cardiac operations an remains relatively constant in the long term even after three years [33], what makes the period of one year after surgery sufficient for HRQOL observation. Despite the higher incidence of complications in the older group, we have found no difference in postoperative HRQOL between the groups age ≤70 years and >70 years except Bodily pain, which improved more in the older group. Same result was also described by the group of Gjeilo [34]. We observed relatively higher values of preoperative SF-36 scores for HRQOL in younger patients, but the differences between preoperative and postoperative SF-36 scores were greater in the older group. This could lead to the conclusion that older patients obtain relatively greater benefit from cardiac surgery than do younger patients in the period of one year after surgery, when most of the postoperative complications have been resolved.

Some of the earlier studies using the SF-36 questionnaire have presented only summaries of the SF-36 scores. For example, they report changes in *physical health status* (derived from *physical functioning, role physical, bodily pain, and general health*) and *mental health status* (derived from *vitality, social functioning, role emotional, and mental health*) [28,29]. In contrast, we have identified the changes in each of the 8 domains of HRQOL and have thus been able to give a more detailed view of the patient’s quality of life. We have used summaries of the SF-36 only in the multivariate analysis, which, in our opinion, is a better and more convenient tool for use as a predictor of postoperative course.

We should comment also on the limitations of this study. These are mainly attributable to the size of the study population (310 patients) and the follow-up period (1 year). Nevertheless, we believe that this study can be the basis for additional research which could prove our conclusions and provide a stronger tool for identifying older people who are likely to experience HRQOL improvement after cardiac surgery. During the study period none of the operative techniques were changed, and that could probably have reduced the potential bias of our longitudinal sample.

## Conclusions

In our opinion, the preoperative HRQOL assessment should be an important part of the preoperative examination, especially in the high-risk patients. Our findings lead us to conclude that older patients with relatively higher cardiac operative risk have lower preoperative HRQOL, but they are more likely to exhibit significant improvement in HRQOL postoperatively. The group of patients age >70 years had more preoperative comorbidities and higher prevalence of postoperative complications, but there was no significant difference in HRQOL in comparison with the younger group of patients 1 year after surgery (except *bodily pain* domain, which improved more in the older group), which was the main finding of this study. If we are able to offer these patients more gentle operative techniques and appropriate postoperative care, then we can achieve not only significant reduction in the number of postoperative complications and mortality but also improvement in their HRQOL.

## Abbreviations

HRQOL: Health-related quality of life; ICU: Intensive care unit; SF-36: Short form 36; CABG: Coronary artery bypass grafting; PCS: Physical component summary; MCS: Mental component summary.

## Competing interests

Financial competing interests

In the past five years, **I have not** received reimbursements, fees, funding, or salary from any organization that may in any way gain or lose financially from the publication of this manuscript, either now or in the future.

**I do not** hold any shares or other securities in any organization that may in any way gain or lose financially from the publication of this manuscript, either now or in the future.

**I do not** hold and **I am not** currently applying for any patents relating to the content of the manuscript**. I have not** received reimbursements, fees, funding, or salary from an organization that holds or has applied for patents relating to the content of the manuscript. I

**I do not** have other financial competing interests.

Non-financial competing interests

**There are no** non-financial competing interests (political, personal, religious, ideological, academic, intellectual, commercial or any other) to declare in relation to this manuscript.

## Authors’ contributions

VK made substantial contributions to the conception and design, acquisition of data, as well as analysis and interpretation of data. AM made substantial contributions to the conception and design, acquisition of data, as well as analysis and interpretation of data. MK made substantial contributions to the conception and design, acquisition of data, as well as analysis and interpretation of data. JČ has been involved in drafting and critically revising the manuscript for important intellectual content. AB has been involved in drafting and critically revising the manuscript for important intellectual content. LP has been involved in drafting and critically revising the manuscript for important intellectual content. VA has been involved in drafting and critically revising the manuscript for important intellectual content. All authors read and approved the final manuscript.

## Authors’ information

The authors are interested in issues of senior-age care in cardiac surgery. In their everyday practice they are striving to improve preoperative conditioning, surgical techniques and postoperative care in order to minimize operative risk for older patients undergoing cardiac surgery.
